# Analysis of Beyfortus^®^ (Nirsevimab) Immunization Campaign: Effectiveness, Biases, and ADE Risks in RSV Prevention

**DOI:** 10.3390/cimb46090617

**Published:** 2024-09-18

**Authors:** Hélène Banoun

**Affiliations:** Independent Researcher, 13001 Marseille, France; helene.banoun@laposte.net; Tel.: +33-6-32-46-78-33

**Keywords:** nirsevimab, Beyfortus, ADE, RSV, bronchiolitis, mAbs, therapeutic monoclonal antibody

## Abstract

Respiratory infections with respiratory syncytial virus (RSV) account for an important part of hospital admissions for acute respiratory infections. Nirsevimab has been developed to reduce the hospital burden of RSV infections. Compared with the product previously used, it has a stronger binding capacity to RSV F protein and a high affinity for FcRn (neonatal receptor for the Fc fragment of IgG), which extends its lifespan. Nirsevimab has been shown to be highly effective in reducing hospitalization rates of RSV infections but a large or unknown number of treated subjects have been excluded in clinical and post-marketing studies. However, analysis of these studies cannot exclude that, in rare cases, nirsevimab facilitates and worsens RSV infection (or other respiratory infections). This could be attributable to antibody-dependent enhancement (ADE) which has been observed with RSV F protein antibodies in inactivated vaccine trials. This risk has been incompletely assessed in pre-clinical and clinical trials (incomplete exploration of nirsevimab effector functions and pharmacokinetics). ADE by disruption of the immune system (not studied and due to FcRn binding) could explain why there is no reduction in all-cause hospital admissions in treated age groups. Given the high price of nirsevimab, the cost-effectiveness of mass immunization campaigns may therefore be debated from an economic as well as a scientific point of view.

## 1. Introduction

Respiratory syncytial virus (RSV) is currently a primary cause of hospitalization in infants and young children globally. Worldwide, it is estimated that 33 million cases of RSV-associated acute respiratory infection (ARI) occur each year in children under 5 [1]. Bronchiolitis can severely affect some children under the age of two and can lead to increased susceptibility to asthma. Hospitalization is required in 3% of cases and admission to intensive care in 2 to 6% of cases [2]. RSV reinfects children annually, and infection occurs despite the presence of maternal antibodies [3]. In infants under 2 months of age, the presence of maternal antibodies and the immaturity of the immune system prevent an effective immune response to an effective RSV vaccine [4]. Infants have a reduced capacity to produce neutralizing antibodies against RSV compared with adults, making the former more susceptible to recurrent infections [5]. In infants, the immune response to RSV is polarized towards a Th2 profile, leading to considerable inflammation of the lungs [6]. The development of RSV vaccines has been hampered by the results of the inactivated vaccine, which has caused deaths from vaccine-antibody-enhanced RSV disease (vaccine-associated enhanced respiratory disease (VAERD), a form of antibody-dependent enhancement (ADE)). The mechanism of VAERD is classically associated with an exaggerated Th2 response, high levels of non-neutralizing antibodies, low levels of neutralizing antibodies, and the presence of eosinophils in the pulmonary epithelium [7]. However, trials of RSV vaccines for children are underway but they do not concern newborn babies: vector-based vaccines studies in children over 12 months of age, with no proven efficacy against RSV infections [8] and with URTI more frequent in vaccinated children [9] and live attenuated virus studies in children over 12 months of age, with no proven efficacy against RSV infections [10] in children over 6 months of age, with an unexpected frequency of respiratory events in the treated groups [11]. Given the low capacity of infants to develop immunity to RSV and past failures in vaccine development, another approach to protecting infants from RSV is the development of monoclonal antibodies, which is passive immunization. Vaccination of mothers has also been shown to be effective: placental transfer of maternal antibodies protects the newborn during the first months of life [12].

Nirsevimab was developed to reduce the burden of RSV infections on hospitals, as its predecessor, palivizumab, which was reserved for at-risk children, required several injections and could not be recommended for all children for economic and logistical reasons. The modification of the Fc fragment of nirsevimab compared with palivizumab enables it to bind more strongly to the Fc neonatal receptor (FcRn), thus prolonging its lifespan and reducing the number of injections to just one. Both monoclonal antibodies are directed against the RSV F protein. Nirsevimab has strong RSV-neutralizing activity. As the search for vaccines has been hampered by the discovery of an ADE effect due to anti-F-protein antibodies, the European Medicines Agency (EMA) has taken into account the theoretical possibility of an ADE through increased FcγR-binding efficacy and has eliminated this risk based on the results of clinical trials and in vitro and animal studies [13] (no ADE has been observed in humans for palivizumab, which has been in use for 20 years [2]).

Nirsevimab was approved by the EMA under a fast-track procedure [13] (p. 7) in accordance with European regulations [14]. This application was prompted by the high estimated burden of RSV lower respiratory tract infection (LRTI) worldwide (3.2 million hospital admissions, including 1.4 million for patients under 6 months of age) [1]. Acute bronchiolitis due to RSV accounts for about 9% of total infant hospitalizations in the USA [15]. This estimate should be compared with the global burden of ARI in the under-5s, estimated at 12 million hospital admissions [16]. Hospitalizations due to RSV in the under-5s would therefore represent around a quarter of ARI admissions. This proportion is consistent with Del Riccio’s 2023 estimate in Europe: admissions for RSV infections account for between 7% and 51% of hospital admissions for ARI (36.1% for France, 33.6% for Luxembourg, and 30.6% for Spain). However, admissions for RSV infection account for only 1.8% to 9.9% (3.7% in France) of hospital admissions in the under-5s, which raises the question of the economic benefits of immunizing all infants. It should be noted, however, that Del Riccio’s estimates are based on incomplete data between 1997 and 2018; various acute respiratory pathologies were grouped together, whether or not they were due to RSV [17]. In France, for example, virological diagnosis is not routinely carried out for all cases of bronchiolitis [18]. Prevention of other respiratory infections must therefore also be taken into account if we wish to relieve the burden on pediatric hospitals. The relative risk of hospital admission for RSV infection has increased in the post-pandemic COVID-19 seasons (2021 and especially 2022); this may be due to an increase in RSV testing. Severe cases increased relatively more in older children (>1 year) during the post-pandemic seasons [19]. If this pattern continues in the future, prevention should target children over one year of age.

The aim of this study is to independently assess the benefit of administering nirsevimab to all newborns to prevent hospital overcrowding, particularly in intensive care units. To be of benefit, nirsevimab must reduce the burden of hospitalization and admission to intensive care for all causes. Analysis of the results of phase 1 and 2/3 clinical trials and immunization campaigns from 2023–2024 will have to look for traces of a possible ADE effect (facilitation of RSV infections), which is part of the EMA’s Risk Management Plan [13]. The non-specific effect of mAbs on a possible disruption of the immune system leading to non-RSV infections must also be examined. We propose a review of the state of knowledge on the mechanisms of ADE in RSV infections. In particular, we show how the pharmacokinetics (role of low initial concentrations) and mode of action of nirsevimab (binding to FcRn and FcγR) are involved in these mechanisms. The shortcomings of pre-clinical and clinical studies in exploring these points are highlighted.

## 2. Clinical Trial Results and 2023–2024 Campaign

### 2.1. Clinical Trial Results

#### 2.1.1. Phases 1 and 2a

In the phase 1b/2a trial on healthy pre-term infants, 5 infants in the treatment group (69 children) developed lower tract respiratory infection (LRTI) corresponding to the clinical criteria used for evaluation, and 3 developed febrile convulsions (but none in the placebo group of 16 children); 1 child in the treatment group was hospitalized for LRTI. With regard to LRTI not corresponding to the protocol definition, 10 children treated versus 1 in the placebo group developed LTRI within 151 days of observation, and RSV A was found 8 days after injection in 2 infants treated with the lowest dose. No RSVs were found in hospitalized children, but no virus tests were carried out in children with LRTI who did not meet the protocol criteria (only severe LRTI requiring assisted ventilation was included) or in those with URTI; no virus tests were carried out, despite the fact that 69% of reported adverse events concerned URTI and that 14.1% of treated children developed LRTI compared with 5.6% of placebo-treated children [20]. In the phase 1 trial in adults, the most frequent adverse event was URTI, with an imbalance in the treated group. Unfortunately, no search for RSV was carried out [21].

#### 2.1.2. Results of Phase 2b and 3 Trials

The trials were sometimes carried out during periods of low RSV circulation; the number of cases observed was low, and that may reduce the statistical power of the trials and the ability to assess ADE [22,23]. Phase 2b and 3 clinical trials on nirsevimab [20,21,22,23,24,25,26] were analyzed by the EMA [27] and Haute Autorité de Santé/French High Authority of Health (HAS) [28].

In the MELODY trial, only LTRIs were evaluated. The 150-day efficacy on hospitalization for LRTI due to RSV is calculated on a very small number of participants (14 hospitalized in all, 0.6% in the treated group and 1.6% in the placebo group, so the absolute reduction risk (ARR) is 1%). Treated participants hospitalized for LRTI due to RSV were hospitalized longer than those in the placebo group, but no significance can be drawn from this, as too few individuals were involved. Additionally, 8.1% (80/987) of treated children were removed from the analysis of results [12].

In the MEDLEY study [24], which combines two studies (one phase 2b [26] and one phase 3 [22]), the ARR of LRTI hospitalization for all causes is 2% (total number 108/2350) and 2% for LRTI due to RSV (28/2350); but 2% of participants in the treated group were excluded before 151 days, compared with 1% in the placebo group, which could distort the estimate of efficacy and safety from the ADE point of view.

In a pre-term infant study, nirsevimab was effective against hospitalization for RSV infection and reduced the severity of RSV infections (of participants hospitalized for RSV infection, all those who were admitted to the intensive care unit (five participants) or received assisted ventilation (four participants) were in the placebo group). The occurrence of LRTI not due to RSV is not affected by treatment. Kaplan–Meier curves show that in the days following the injection, all-cause LRTIs are equally frequent in both groups; the protective effect does not appear until 20 or more days after injection. This does not rule out an early ADE of nirsevimab. All-cause hospitalizations are not reported in this study. In addition, 5.79% (56/968) of treated children were withdrawn from the study [26].

The open-label HARMONY study [25] involved 8058 infants under 1 year of age in Europe from 2022–2023. The investigator was sometimes the treating physician and decided whether or not hospitalization was due to RSV. RSV PCR tests were not routinely performed, and only after hospitalization, and 16 hospitalized children did not undergo RSV PCR tests. Final results have not been published, as 12 months’ follow-up is required for all children. The study showed a 1.2% reduction in the ARR of hospitalization for LRTI due to RSV and a 0.4% reduction for very severe LRTI. For LRTI of all causes, the ARR of hospitalization was 1.3%. The Kaplan–Meier curves differ from country to country, and no explanation is given; in France, hospitalizations for LRTI due to RSV occur from the first day of the study among placebos, whereas in Germany they begin only 15 days post-injection and 1 month later in the UK. This may reflect the staggered circulation of the virus in these countries. Among grade 3 serious adverse events, there were as many infections in the treated group as in the placebo group and more severe infections in the treated group than in the placebo group. Among treated children, only 500 out of 4037 (12%) were less than 7 days old. Additionally, 0.22% of treated children were withdrawn from the study. The main results of the clinical studies are summarized in Table 1.

#### 2.1.3. Deaths in Trials

The FDA notes an imbalance of deaths in favor of the treated group: 12 deaths in the 3710 treated participants (0.32%) versus 4 in the 1797 control participants (0.22%), taking into account 1 death in the placebo group 6 days after the end of the study and without specifying deaths in the treated groups after the end of the study [30]. In the studies listed by the EMA [27], eight deaths were recorded (with the same percentage—0.3%—in the treated and placebo groups). The Domachowske study on fragile and pre-term infants [20] (which was published as a correspondence, i.e., not peer-reviewed) concerns premature babies and newborns suffering from heart or lung disease and compares the effect of the monoclonal antibody previously used (Synagis^®^, palivizumab) with Beyfortus^®^ (nirsevimab). It provides a full description of the deaths observed: of the six deaths listed, five were related to pneumonia or bronchiolitis not attributed to treatment (five babies who died were treated with nirsevimab (5/614 = 0.81%) and one (1/304 = 0.32%) with palivizumab). Despite the low number of deaths, there was an imbalance against nirsevimab. In the absence of a placebo group, this result may be interpreted in terms of the lower efficacy of nirsevimab compared with palivizumab. It is also possible that nirsevimab facilitates and aggravates bronchiolitis; these injections take place during periods when the virus is circulating. In particular, nirsevimab appears to be more dangerous than its predecessor, palivizumab. RSV testing was not performed except in one case (negative result), which is regrettable in a study of the efficacy of a product against RSV. No autopsies for histopathological analysis of the lungs were reported although the EMA recommends the investigation of immunopathological phenomena after any immunization failure [27]. It was therefore impossible to look for signs of inflammation and VAERD. Additionally, 6.86% of treated children (63/918) were withdrawn from the study [20]. In conclusion, it is therefore impossible to exclude ADE as a cause of these five deaths.

### 2.2. Phamacovigilance Data and Results of the 2023–2024 Season Immunization Campaign

The EudraVigilance database records spontaneously reported adverse events for nirsevimab. As of 15 April 2024, there were 140 reports, mainly from healthcare professionals (138/140), 89 of which were for “bronchiolitis”, 129 for “RSV”, and 56 for “drug ineffective”. Only 26 reports did not concern respiratory events; the most frequently reported adverse event is bronchiolitis and could be due to an ADE effect [31]. Moreover, according to the Île de France pharmacovigilance center, there is a theoretical risk of aggravation of RSV infection through non-neutralizing antibodies [32].

Following its approval by the EMA on 31 October 2022 [27] and by the FDA on 17 July 2023 [33], nirsevimab was recommended for infants and children under 2 years of age in four countries for the 2023–2024 season: the USA, France, Spain, and Luxembourg. In the USA, on 3 August 2023, the Advisory Committee on Immunization Practices recommended nirsevimab for infants aged < 8 months born during or entering their first RSV season and for infants and children aged 8–19 months who are at increased risk of severe RSV disease entering their second RSV season [34]. In France, the HAS recommends nirsevimab for infants born at a gestational age of 35 weeks or under and under 6 months of age entering their first RSV season, infants under 2 years of age having required treatment for bronchopulmonary dysplasia in the past 6 months, and infants under 2 years of age with congenital heart disease with a hemodynamic impact [28]. In Spain, the Spanish Society of Neonatology recommends nirsevimab for all newborns and at-risk children under two years of age [35]. In Luxembourg, the government has recommended immunization for all newborns and children under one year of age [36].

#### 2.2.1. USA Results of Immunization Campaign

In the USA, immunization coverage for children under 20 months of age is about 20%, but data do not include children born after 1 October 2023 [37] (making it unnecessary to compare hospitalization rates for RSV and non-RSV LRTI with previous seasons). An exceptional peak in the rate of hospitalizations due to RSV was observed from 2022–2023 in 0–4 year olds [38]. The results of this campaign are set out in a non-peer-reviewed article by the CDC [39]: a 90% effectiveness rate against RSV-associated hospitalizations is announced, but the method of calculation is not set out (this is a case–control study). But it is impossible to match a case with a control given the disproportion of the groups (59 children treated with nirsevimab out of 699); the most optimal case-to-control ratio is 1:1 and statistical power can be improved by increasing the ratio up to four controls/one case [40]. Participants were infants under 8 months of age hospitalized for ARI, with known nirsevimab status and having been tested for the presence of RSV by PCR. Hospitalizations less than 7 days after nirsevimab injection are excluded. The published figure shows that ARIs are more frequent in the days following injection, and it is unfortunate that the first 7 days have been excluded from the calculation (20 nirsevimab-treated children are excluded for this reason, reducing the number of treated children studied to 59). Of the children hospitalized for ARI due to RSV, 6 had received nirsevimab, and, if we were to add these 20 excluded children, the number would rise to 26, and the product’s effectiveness would be less than the advertised 90%. It is therefore impossible to evaluate the possible ADE, but we can suspect that it exists, since of the total number of hospitalizations due to RSV, 77% (20/26) are observed within 7 days of injection [39].

#### 2.2.2. Luxembourg Results of Immunization Campaign

In Luxembourg, nirsevimab immunization began in maternity hospitals on October 1, 2023, with 84% calculated coverage. Children hospitalized with respiratory symptoms are systematically tested for RSV. RSV-positive newborns under 6 weeks of age are routinely hospitalized, so hospitalization is not a criterion of severity for this age group. As compared with the 2022 season, the reduction in hospitalizations for RSV in children under 5 years of age is lower than in those under 6 months of age, and the reduction in the burden on hospitals may be lower than hoped [41], since severe cases are more frequent among children over 1 year of age [19]. No data have been published on all-cause hospitalizations and hence on the potential reduction in hospital burden. In 2023, there was a 38% decrease in the number of children under 5 hospitalized for RSV infection (88,4% were not immunized) and a 69% decrease in those under 6 months (65.3% not immunized) as compared with the 2022 season. As in other countries, the number of hospitalizations among young children was high in 2022. So, the reduction in the number of children under 5 hospitalized for RSV infection cannot be attributed to immunization with nirsevimab [41].

#### 2.2.3. France Results of Immunization Campaign

In France, approximately 230,000 doses of nirsevimab were administered between 15 September and 15 December 2023 for about 186,000 births (the remaining 44,000 doses had to be administered as catch-up doses to older children) [42]. Indeed, 42,290 doses of nirsevimab were administered between 15 September 2023 and 31 January 2024 to children born between February 6 and 15 September 2023 [43]. We can therefore assume that immunization coverage is high in France for this season. According to Santé Publique France [18], in comparison with previous seasons (with the exception of the 2022–2023 season, which was exceptionally intense as in the other countries), bronchiolitis-related activity was comparable in intensity in primary care, while hospital activity was comparable to or slightly higher than the reference seasons. The proportion of intensive care unit admissions for children under 2 years of age was comparable to the reference seasons. In infants aged 3 months and over, emergency room visits and hospitalizations were higher than in previous seasons and close to levels for the 2022–2023 season. This activity is even higher at the start of the epidemic (weeks 37 to 46, i.e., from 15 September 2023, the start of the immunization campaign). The same type of data (exact number of intensive care unit visits for bronchiolitis) is not available for previous seasons (only the number of hospitalizations is provided [44]). RSV was systematically tested for in hospitalized patients only during the 2023–2024 season. So, it is impossible to derive from these data an estimate of the effectiveness of nirsevimab on the risk of ICU hospitalization for RSV.

A case–control study in preprint surveys children under 5 months of age admitted to intensive care for bronchiolitis between 15 September 2023 and 31 January 2024 in France [45]. The effectiveness of nirsevimab against ICU hospitalization for RSV bronchiolitis was estimated at between 74.4% and 80.6%, depending on the inclusion criteria. Data are from Santé Publique France but 52% of cases are excluded. Two hundred and thirty-eight cases (newborns hospitalized for bronchiolitis with a positive RSV test) were compared with fifty controls (newborns hospitalized with a negative RSV test). It was therefore impossible in this study to match each case with a control, given the disparity in the numbers of cases and controls. The majority of these 52% of excluded cases therefore concerned infants with missing data, which is a pity, since according to SPF’s 2023–2024 report [18], surveillance of RSV infections in patients admitted to the intensive care unit usually concerns only those over 18 years of age. For the 2023–2024 season, non-exhaustive surveillance is carried out for all cases of bronchiolitis in patients under 2 years of age, regardless of the virus involved (identified or not). It should be noted that for the 2020–2021 and 2021–2022 seasons described by Vaux [46], a case of bronchiolitis in a hospitalized child under 2 years of age is defined [47] according to the ICD [48] and corresponds to codes J21, J21.0, J21.8, J21.9, which all concern acute bronchiolitis regardless of the virus involved. Concerning the possible detection of early ADE among nirsevimab-treated newborns, comparison of the main analysis and sensitivity analysis 2 shows that 45% (17/38) of PICU admissions for LRTI not due to RSV and 27% (14/51) of LRTI cases due to RSV occurred less than 8 days (or after an unknown delay) after nirsevimab injection. Among nirsevimab-treated newborns, a comparison between sensitivity analyses 1 and 2 shows that 8% of PICU admissions for LRTI not due to RSV and 8% for LRTI due to RSV definitely occurred less than 8 days after nirsevimab injection. These data cannot rule out ADE from RSV infection or other infections (due to immune system disruption) [45].

A peer-reviewed case–control study indirectly calculates the effectiveness of nirsevimab against RSV bronchiolitis; depending on the different calculation methods, this is around 80%. But 17% (142/832) of children hospitalized for RSV infection were excluded from the study. The calculation of effectiveness against severe RSV infections is incomprehensible (last line and legend of Figure 2 are contradictory [42]). In the intention-to-treat analysis (including all patients regardless of the date of nirsevimab injection), there was no effectiveness during the first 7 days (19% of cases—15/75—and 20% of controls—23/120—received nirsevimab). This lack of efficiency is comparable with the advertised 80%. This does not rule out early ADE. Here again, the number of controls is less (by half) than the number of cases and does not allow a correct calculation. Recruitment was limited to six French hospitals and does not appear to be representative, since only 8.7% of cases and 28.1% of controls were treated with nirsevimab; we should observe much higher coverage for children with a median age of 3 months. In fact, 230,000 doses were administered during the study period for around 186,000 births [42].

#### 2.2.4. Spain Results of Immunization Campaign

According to an October 2023 survey, immunization coverage is above 80% in almost all regions of Spain [49]. This is confirmed by more recent publications (90% in Andalusia [50], 96.5% in Asturias [51], and 86% in Madrid [52]). At a national level, according to the SIRVA network [53], hospitalization rates for RSV infection in 0–4 year olds are comparable for the 2022–2023 and 2023–2024 seasons (the peak is 140/100,000). In Galicia, according to the regional Ministry of Health report, epidemic waves of RSV infection are comparable over time between 2022–2023 and 2023–2024 in terms of positivity for RSV in the general population. There has been a drastic reduction in hospitalizations for RSV in both newborns and children born since April 2023, with a high rate of immunization coverage (93% in NN and 86% for catch-up infants born before the campaign). For children entering their 2nd RSV season, hospitalization rates are identical to previous seasons (they have not been immunized) [54].

The first results of the estimation of the effectiveness of nirsevimab on newborns, children under 6 months, and at-risk children under 2 years of age, in Galicia, between 25 September and 31 December 2023, have been published [55]. This study is scheduled to run until 2026 [56], so the publication only concerns results from the first 3 months of the immunization campaign. Nirsevimab was offered to all newborns from 25 September 2023 unless medically contraindicated and to all children born after 1 April 2023. The reasons for non-immunization (parental refusal or medical contraindication—fragile state of health of the newborn) are not precisely known; some of the non-immunized newborns were certainly more fragile than those immunized, and comparison between the two groups will be difficult as they do not involve equivalent populations in terms of health status. Given the small number of non-immunized infants, case–control matching is impossible. Hospitalization for RSV with severity criteria occurred only in the treated groups which may be a sign of treatment-induced ADE. Nosocomial infections were excluded. Nirsevimab is administered on the day of birth in Galicia. The study’s lead author confirms that all infections developed in hospital for whatever reason were excluded (personal communication); this means that newborns who acquired the infection during their stay in the maternity hospital were excluded from the study. The estimated effectiveness should have taken into account these excluded cases, the number of which is not provided. Moreover, these “nosocomial” cases can be explained by early post-injection ADE of nirsevimab, whose exclusion does not allow us to evaluate. The effectiveness of nirsevimab against hospitalization for RSV infection was 82% (in 46 cases) in intention-to-treat analysis on children under 6 years of age with no risk factors. Sensitivity analysis excludes breakthrough infections and gives 87.5% effectiveness (in 44 cases). For all-cause hospitalization, effectiveness was 66.2% (in 366 cases). The authors compare the results of the 2023–2024 season with previous seasons since 2016, excluding the 2020–2022 season because the bronchiolitis epidemic was smaller than normal, but do not exclude the 2022–2023 epidemic, which was larger. According to Figure 4 in the Appendix [55], if we exclude the exceptional 2022–2023 season, the 2023–2024 hospitalization rate is comparable to previous seasons, albeit slightly lower [55]. So, the effectiveness of nirsevimab in reducing all-cause and RSV hospitalization rates is questionable.

In Navarre, the effectiveness of nirsevimab was calculated from epidemiological surveillance of newborns between October 2023 and January 2024 (the RSV circulation season) according to their immunization status (92% of newborns were treated, mostly within the first 7 days of life). Despite the low number of non-immunized children, effectiveness could be calculated based on emergency room visits for RSV (87.8%), hospitalization for RSV (88.7%), and admission to intensive care (85.9%). No details are given on the clinical results of the five children admitted to intensive care (two non-immunized and three immunized). It is not known whether immunized children developed more or less severe infections than untreated children. It should be noted that newborns injected less than 1 day previously are counted as non-immunized (if they develop RSV infection within 24 h of injection, early ADE cannot be assessed) and children admitted for respiratory illness not due to RSV were not counted. It is therefore impossible to assess the ADE of RSV and other viral infections and the effectiveness of nirsevimab on all-cause hospitalizations [57]. Data from previous years are not comparable [58]. The main results of post-marketing campaigns are summarized in Table 2.

### 2.3. Neonatal Deaths in France

**Figure 1:**
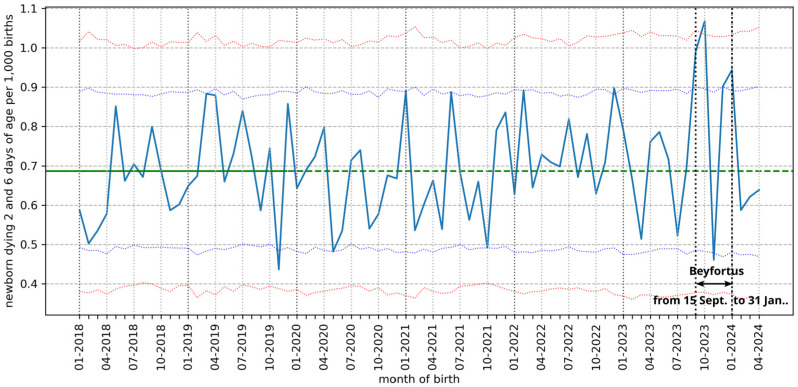
Rates of death per month between 2 and 6 days of life per 1000 births in France (from April 2018 to April 2024). The time window of the nirsevimab immunization campaign (Beyfortus©) is displayed. For each month, newborns born during the month and dying between 2 and 6 days of age are counted. The mortality rate is obtained for each month by dividing the number of deaths by the number of births. The reference rate is calculated by dividing the total number of deaths by the total number of births in 2018 and 2019 (before the COVID-19 pandemic): it is 0.69 deaths between 2 and 6 days of life per 1000 births. This rate is indicated by the green horizontal line. For each month, the expected death rate is calculated by multiplying the reference rate by the number of births. Poisson’s law (a statistical law used for rare events) was used to calculate the different confidence intervals for mortality rates at 95% (classic threshold) and 99.8% (alarming threshold). The 95% confidence interval is marked by the blue dotted lines, with a probability of less than 2.5% for rates below or above the lines at the bottom and top, respectively. The 99.8% confidence interval is denoted by the red dotted lines with a probability of less than 1% for rates below or above the lines at the bottom and top, respectively. The black dotted vertical lines represent the month of September 2023, with the nirsevimab immunization campaign starting on the 15th of that month, and the month of January, with the last doses administered (the end of the nirsevimab distribution campaign in France was announced for December 2023 [64]). Monthly numbers of births and deaths are taken from Institut National de la Statistique et des Études Économiques (INSEE) data [61,62].

In France, the government recommends injecting nirsevimab into all newborns leaving the maternity hospital [59]. The average length of stay for childbirth is 4.6 days [60]. Based on official French data on deaths [61] and births [62], it is possible to calculate the mortality rate between 2 and 6 days of life during the nirsevimab immunization campaign and to compare it with the periods before and after (Figure 1). There is a highly significant peak (*p* = 0.006) for babies born in September 2023 and dying between 2 and 6 days, with 55 deaths (95% CI 27–50). And there is a very highly significant peak (*p* = 0.0007) for babies born in October 2023, with 62 deaths (99.8% CI 22–60). In November 2023, after the two highly significant peaks, we observe a significant drop in the number of deaths (*p* = 0.02), with 26 deaths (95% CI 27–50). In December 2023, the number of deaths is high, at the limit of significance at 2.5%, with 50 deaths (95% CI 26–50). And in January 2024 the number of deaths is significant (*p* = 0.022), with 52 deaths (95% CI 27–50). In February, March, and April 2024, mortality rates between 2 and 6 days of life returned to normal. Apart from the month of November with a significantly low rate of deaths, the rate of babies dying between 2 and 6 days of age is significantly higher than the rate expected between September 2023 and January 2024, the period of RSV circulation and nirsevimab immunization. Doses of nirsevimab quickly ran out in October [63] and dispensing of doses distributed outside maternity wards was drastically reduced in November, only to resume in early December 2023, with the last doses administered in January 2024 [43]; it is therefore possible that dispensation in maternity units was also reduced in November 2023, this being in line with the low rate of deaths between 2 and 6 days of life compared with other months. According to the data [18], the bronchiolitis epidemic was less intense in November 2023, which could explain the dip in neonatal mortality, although this does not rule out the possible role of an early ADE, since for this to manifest itself, the virus must be circulating strongly. It should be noted that the latest data are not yet fully consolidated. An early ADE of nirsevimab cannot therefore be ruled out to explain why these significant peaks in neonatal mortality coincide with the immunization campaign.

**Figure 2:**
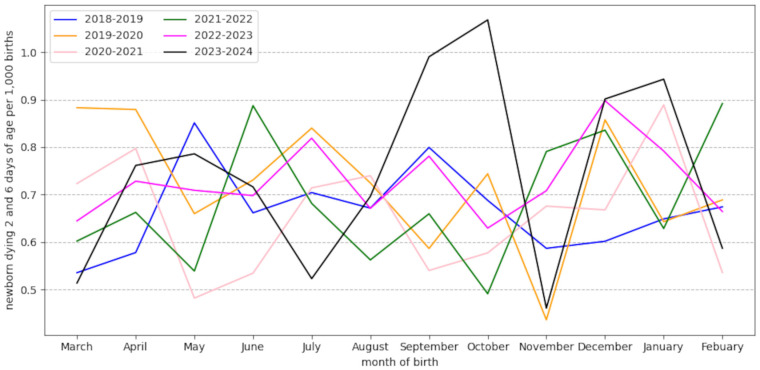
Rate of deaths per month between 2 and 6 days of life per 1000 births in France between March 2018 and February 2024: superposition of the curves obtained for each year. Between March 2018 and February 2024 the monthly rate of deaths between 2 and 6 days of life per 1000 births was calculated according to INSEE data [61,62]. Data have only been collected since 2018, the year for which the early neonatal mortality stabilized (after a drop between 2001 and 2011 and an increase between 2011 and 2018) [65].

Superimposing the neonatal death rates for each month between 2018 and 2024 highlights peaks in deaths that fall outside the average range and recur 2 months in a row (September/October and December/January) for the 2023–2024 season; the sharp drop in November is also found in 2019 (excluding the COVID pandemic and the nirsevimab immunization season) (Figure 2).

It is impossible to perform a time series analysis, as monthly data on the number of doses of nirsevimab administered are not available; we only know the start and end of the immunization campaign and can only assume that the number of doses administered in hospitals in November 2023 fell drastically. It is therefore impossible to establish a causal link between the administration of nirsevimab and the observed spike in neonatal deaths; however, the biological plausibility of this link is demonstrated by the review of the biological mechanisms of ADE and is suggested by the results of clinical and post-marketing studies. The dip in the November mortality rate is observed not only in 2023 but also in 2019 and cannot therefore be explained only by a possible shortage of nirsevimab doses. This confirms that a causal link between the immunization campaign and the variation in death rates cannot be established with the data currently available. However, the repetition of two peaks of excess mortality (each lasting 2 months) could be a signal to watch out for. Between March 2018 and February 2024 the monthly rate of deaths between 2 and 6 days of life per 1000 births was calculated according to INSEE data [61,62]. Data have only been collected since 2018, the year for which the early neonatal mortality stabilized (after a drop between 2001 and 2011 and an increase between 2011 and 2018) [65].

## 3. Design of Nirsevimab

**Figure 3:**
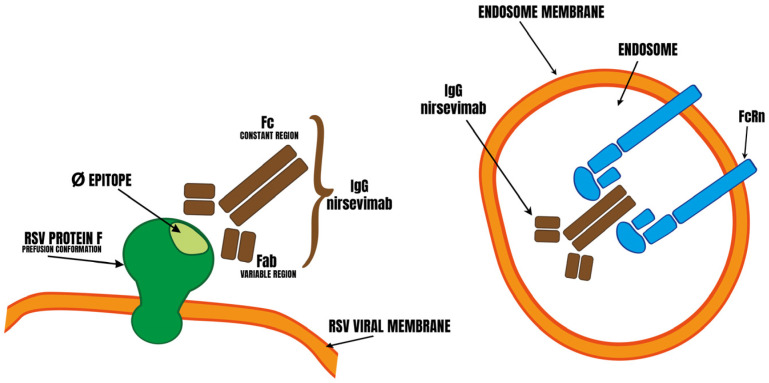
Function of nirsevimab. Left: nirsevimab binds strongly to epitope Ø of the RSV fusion protein in prefusion conformation [4]. Right: after endocytosis, YTE mutations on the Fc fragment of nirsevimab give it a high affinity for FcRn, protecting it from degradation in the acidic environment of the endosome.

Palivizumab, mAb against RSV F protein (viral–cell membrane fusion protein) reduced hospital admissions in observational studies [2] but a Cochrane review [66] showed no beneficial effect of palivizumab compared with placebo. Pavilizumab should be administered once a month during the RSV circulation season, due to its short serum half-life [67]. Manufacturers of anti-RSV mAbs have made efforts to increase the half-life and affinity for F protein [4] (Figure 3 left). Nirsevimab is derived from this research; it has superior neutralizing power to pavilizumab and an extended half-life thanks to YTE mutations (80–120 days) [2]. YTE mutations increase FcRn binding (the neonatal Fc crystallizable fragment receptor (FcRn) binding to the Fc portion of IgG) [68]. FcRn protects IgG from intracellular degradation by a pH-dependent recycling mechanism and enables its transport across cell barriers. IgG ingested by the cell via pinocytosis binds to FcRn in the acidic environment of the endosome (Figure 3 right) and is transported intact back to the surface of the plasma membrane [69]. YTE mutations increase mAb affinity for FcRn at acidic pH [4] and nirsevimab binds FcRn with very low affinity at neutral pH [70].

## 4. Definition, Role, and Localization of FcRn: Based on Our Knowledge of the Role of FcRn, What Might Be the Consequences of a mAb’s Increased Affinity for This Receptor?

### 4.1. Transport of Free and Antigen-Bound IgG

The FcRn is a multifunctional atypical Fc-gamma receptor (FcγR). IgG binding to FcRn accounts for its long life (3 weeks) [69]. FcRn enables IgG to be transported across cell barriers, including that of the lungs. FcRn binds IgG only in acidic environments (pH between 5.0 and 6.5). FcRn is expressed on cell surfaces and in cell endosomes in mucous membranes (epithelium of placenta, liver, kidneys, genital tract, lungs) and in endothelial and hematopoietic cells [71,72]. In the lungs, IgG is transcytosed through the respiratory epithelium from the lumen to the serosa by FcRn [68,69]. At these sites, FcRn ensures IgG transport across the mucosal barrier and may play a role in immune surveillance and host defense [73]. FcRn enables bidirectional transcytosis of IgG in the apical to basal and reverse direction [70,74,75,76]. IgG preferentially binds to FcRn on the surface of mucous membranes when these are acidic; this is the case in the intestine [77], which allows newborns to acquire IgG from breast milk. But the neonatal lung mucosa has a variable pH and can be acidic in some individuals [78,79,80,81].

Antigen-bound IgG (IgG immune complex (IgG-IC)) also binds to FcRn [82] and cause IgG-ICs to enter cells [69,70]. This transport does not require transepithelial pH gradients established at epithelial surfaces [72]. An antigen bound to the Fc fragment allows FcRn-specific transport across the mucosal barrier [83].

### 4.2. Intracellular Transport of Viruses by FcRn

Echovirus not bound to antiviral IgG can enter target cells by FcRn binding [73,84,85]. Concerning the viruses linked to specific antibodies, a monoclonal antibody against a reovirus could increase virus growth in a murine macrophage-like cell line; this increase occurred with sub-neutralizing antibody concentrations and was mediated by the macrophage-like cell Fc receptor. No specific reference was made to FcRn, which was not characterized at the time (1983) [86]. FcRn can promote the entry of IgG–virion complexes (cytomegalovirus (CMV)) by transcytosis across the placenta. It has been suggested that IgG–virion complexes can be transcytosed in other tissues: intestine, kidney, lung, and breast. However, this only occurs with low-affinity antibodies (or low levels of neutralizing antibodies) [87]. Highly neutralizing monoclonal antibodies increased HIV transcytosis in a human cell line (and more strongly at acidic pH), with this transcytosis depending on the FcRn of the target cells [88]. Strongly neutralizing antibodies do not confer protection in challenge assays and ADE may be an FcRn-mediated mechanism [89].

The enhanced binding of nirsevimab to FcRn therefore extends its lifespan, but when bound to RSV, it is not impossible that this binding facilitates entry of the virus into certain cells, particularly in the lower respiratory tract, facilitating or aggravating infection. FcRn is expressed in macrophages [82]. The lungs are an important site of FcRn expression in several species, and FcRn is highly expressed by alveolar macrophages of all species [68].

## 5. What Are the Mechanisms of ADE in Viral Infections and Following Antiviral Vaccinations, and How Might an RSV F Protein mAb with Increased Affinity to FcRn Be Involved? Could mAb Binding to Other FcγRs Be Involved?

### 5.1. Mechanisms of ADE of Viral Infections by Specific Antiviral Antibodies

ADE has been described in numerous viral infections and antiviral vaccinations (arthropod-borne viruses, alphaviruses, flaviviruses, respiratory viruses such as influenza virus, coronaviruses, RSV, Ebola [90], and measles virus [91]) and has been attributed to sub-neutralizing concentrations of antibodies capable, under certain conditions, of enhancing viral infection by facilitating viral entry into target cells. ADE is produced by interaction between immunoglobulin Fc fragment receptors (FcγRs) or complement receptors and the virus–antibody complex. Extrinsic ADE promotes infection of myeloid cells (monocytes, macrophages, dendritic cells, and granulocytes). Internalization of virus–antibody complexes can also modulate the expression of the innate cytokine response to the virus, which is intrinsic ADE [90]. The binding of antibodies to cellular FcγRs and to complement can also lead to a more global disruption of the immune system.

### 5.2. ADE during Infections with and Vaccinations for RSV or Other Viruses

ADE is observed during infections with viruses that replicate preferentially in macrophages [92]. Severe RSV infections are observed in the first 6 months of life when maternal antibodies are still circulating. Infants with maternal antibodies to RSV were not only susceptible to RSV infections but the rate of severe illness was higher in these infants than in infants without maternal antibodies [92,93]. Antibody levels do not correlate with protection against RSV infection in challenged animals [93]. However, the severity of RSV infection requiring oxygen therapy is lower in breast-fed infants than in formula-fed infants [94]. Anti-RSV IgG is found in 95.2% of newborns at birth; maternal antibodies decrease considerably after 2 months of life and the lowest level is reached at 7 months, after which the level rises again with the first infections. The hospitalization rate for newborns peaks at around 6 months of age. Maternal antibodies do not protect against reinfection due to the short lifespan of the antibodies and also to mutations in the virus, which escapes the antibodies acquired by the previous infection [95]. These aggravated RSV infections could be due to inflammatory cytokines produced during macrophage infection [96]. This was confirmed in a re-examination of the case of two young children who died of RSV disease after being vaccinated with an inactivated vaccine in a trial (80% of children vaccinated and subsequently infected with RSV had to be hospitalized). VAERD-associated bronchiolitis is characterized at autopsy by diffuse inflammation of the alveoli and infiltration by neutrophils and eosinophils. Eosinophilia is not found in the blood. In classical bronchiolitis, inflammation is centered on the bronchioles, and eosinophils are virtually absent. In VAERD, unprotective antibodies form immunocomplexes with complement, triggering a Th2 polarization of the cellular response (Th2 cytokines increase bronchoconstriction and lung pathology) [97].

### 5.3. Role of Complement in ADE (RSV and Other Viruses)

Complement is expressed by many cell types on their surface, and its binding to the virus–antibody complex can facilitate viral entry into cells [98,99]. In influenza virus infection, autopsies of fatal cases have shown large amounts of deposition of a complement component activated by the virus–antibody immune complex [100]. Complement-fixing immune complexes play an important role in enhanced RSV disease; IgG and complement are colocalized in the alveoli and bronchioles of mice suffering from VAERD [101].

### 5.4. Role of IgG Binding to FcγR in ADE during RSV Infection

In humans, there are three types of Fc receptor that bind human IgG. FcγRI is present exclusively on monocytes/macrophages and binds human IgG with high avidity. FcγRII and FcγRIII are found on monocytes, macrophages, eosinophils, neutrophils, natural killer cells, B lymphocytes, and T lymphocytes. Both receptors have relatively low specificity and relatively low avidity for IgG compared with FcγRI [92]. The binding of FcγRs (type I or II) depends on the conformational state of IgG (open or closed) [5]. The role of FcγRIIIa is said to be double-edged. FcγRIIIa in NK cells participates in viral clearance by eliminating infected cells through the antibody-dependent cell-mediated cytotoxicity (ADCC) phenomenon and may play an anti-inflammatory role during RSV disease [102,103]. But FcγRIIIa expression is increased in NK cells from patients with severe RSV-associated pathology, thus suggesting that this receptor may contribute to RSV disease. In mice, FcγRIII has a proinflammatory role in RSV infection. FcγRIII is present on nasal epithelial cells and controls the balance between tolerance and inflammation through its interaction with TLR4 [102].

### 5.5. Involvement of the Monocytic Lineage in ADE (in Case of Viral Infection and after RSV Vaccination)

The involvement of the monocytic lineage, via the FcγRs it expresses, has been shown in ADE during dengue [104] and for other viral infections [105]; macrophages can promote viral replication. Macrophages and bronchial epithelial cells are susceptible to RSV replication [3,106,107,108] and are involved in ADE during RSV infection [3,92,96,109,110,111]. Severe post-vaccination RSV infections may also result from increased viral replication in macrophages [110]. Serum from immunized children is rich in RSV F-protein antibodies and is capable of aggravating infection of a macrophage line [3,111] or pulmonary dendritic cells in vitro [112], this effect being Fc-receptor-dependent. 

### 5.6. Immune System Disruption Caused by IgG Binding to FcRn

FcRn located in the lungs could cause non-specific aggravation of infections under inflammatory conditions. FcRn is expressed in lung parenchyma and lung immune cells and enables preferential secretion of low-affinity IgG into the lumen. IgG1 with high affinity for FcRn could saturate FcRn receptors locally, counter-intuitively allowing preferential passage of low-affinity IgG into the pulmonary lumen. Under these inflammatory conditions, this process could damage lung tissue [113]. The FcRn binding of IgG-ICs formed in the presence of excess antibody has an immunosuppressive effect, in contrast to those formed in the presence of excess antigen (which are immunogenic) [77]. IgG-antigen ICs can activate tissue factor (TF) via monocyte FcRn; this activation of TF leads to the activation of FXa, which is involved in thrombotic phenomena. In this process, FcRn and FcγRIIa may cooperate [114].

IgG-IC binding to FcRn has an inflammatory role through FcRn engagement on the cell surface in conjunction with binding to other FcγRs; this process could contribute to autoimmunity phenomena [115].

### 5.7. Known ADE with mAbs against RSV and Other Viruses

The therapeutic effect of mAbs may result from binding to complement components. Since C1q- and FcγR-binding sites on the Fc domain are proximal and partially overlapping, amino acid substitutions altering FcγR binding also alter C1q recruitment and vice versa [116]. Binding mAbs against Ebola virus can worsen the infection. It is necessary to completely (and not partially) eliminate the effector functions of mAbs to obtain a therapeutic effect; these effector functions could induce an ADE effect which would cancel out the therapeutic effect [100]. Regarding RSV, in cultured macrophagic cell lines, neutralizing anti-F-protein mAbs induce ADE at low concentrations [111,117]. The extent of ADE correlates with complement fixation and the level of neutralizing antibody and can vary in the presence of antibody mixtures [117]. This has recently been confirmed and attributed to IC deposition [105,109].

### 5.8. Importance of Antibody Levels and Quality: ADE Can Occur in the Presence of Low Levels of Strongly Neutralizing RSV Antibodies

In the case of mAbs capable of neutralizing dengue virus, ADE occurs only with certain epitopes and viral strains. For the same antibody, neutralization occurs at low dilution and ADE at high dilution; neutralization occurs when virions are in the presence of a large excess of antibody [104]. In the same way, ADE induced by antidengue vaccine antibodies occurs only at low antibody levels [118]. An in vitro ADE effect has been demonstrated on cells expressing certain FcγRs with sub-neutralizing antibody concentrations in certain viral infections [100]. Nirsevimab has a higher neutralizing capacity than palivizumab, which does not always prevent ADE [12,87,88,89,109,110].

We have seen above the possibility of severe RSV infections in children with low levels of circulating maternal antibodies. The effect of antibody concentration on the ability to induce ADE in vitro has been shown for the anti-RSV-F-protein mAb palivizumab and the precursor to nirsevimab (D25, lacking the YTE mutation that increases affinity for FcRn): they induce ADE at low concentrations, whereas they are strongly neutralizing; at high concentrations, infection is blocked. This in vitro ADE was shown for palivizumab on a human monocytic cell line. For D25, maximum infection was observed at a dilution corresponding to a concentration as low as 1 ng/mL. D25 induced a stronger ADE effect than palivizumab when binding to the FcγR of cells; the ADE effect outweighed the neutralizing power [93]. Palivizumab recognizes the post-fusion (and pre-fusion) form of F protein, whereas D25, which is more potently neutralizing, is specific for the post-fusion form, as is nirsevimab [7]. It cannot therefore be ruled out that nirsevimab may also induce a low-concentration ADE like D25, since it has the same structure as D25 with the added YTE mutations. Van Mechelen’s experiments at low concentration should be repeated with nirsevimab: does nirsevimab induce a stronger ADE than palivizumab at low concentrations?

## 6. How Were the Factors Likely to Cause ADE with Nirsevimab Assessed?

No ADE has been observed in humans for palivizumab, which has been in use for 20 years [2]. But all the modifications of nirsevimab compared with palivizumab theoretically open up the possibility of an ADE via binding to FcRn or other FcγRs [71,116,119].

### 6.1. Pharmacokinetics

Given the possibility that a low level of neutralizing antibodies may be capable of inducing ADE, it is important to be familiar with the pharmacokinetics of nirsevimab.

Animal studies [4,70] are carried out with doses well above those used in clinical trials and for commercial use. One study is IV and with other mAbs similar to but different from nirsevimab. The results show a rapid decline (within 24 days) in serum and much lower concentrations in nasal fluid (1/10,000) and bronchoalveolar washings (less than 1/1000), as well as wide inter-individual variations. YTE mutations halve the bronchial bioavailability of mAbs [70]. The EMA report [27] points out that, in ADE trials, rats treated with nirsevimab are exposed to RSV at the time of peak antibody concentration. It is therefore possible that, in animals, sub-neutralizing concentrations are present in the lungs in the first few weeks after injection.

Clinical studies in adults [21] and in premature infants with an average age of 6 months [20] show that peak serum concentrations are reached in 3 and 8 days, respectively; in adults the peak neutralizing activity is reached 6 days after IM. Furthermore, 5% of children have an antibody level below the effective target concentration (4-fold below the baseline of neutralizing antibodies) after 8 days and 10% after 151 days. Antidrug antibodies (ADAs—targeting YTE mutations) are detected in 28% of treated subjects; these ADAs could impact pharmacokinetics between days 151 and 361 in some subjects [20,24]. Results from other clinical trials also show that rarely, in children, the theoretical threshold of neutralizing activity is not reached or is only reached after 150 or 200 days [22,24]. Pharmacokinetic studies have not been carried out in healthy neonates of a few days old, who represent the target population for the 2023–2024 campaign. In clinical trials, children were at best around 3 months old; in particular, in the open-label study which included a large number of participants (4037 treated), only 12% were less than 7 days old [25]. The pharmacokinetics at birth may differ from those observed in infants of a few months old, but the recommendations concern newborns in the first few days of life (on leaving the maternity ward in France [59], on the first day of life in Galicia [55], in the first 7 days of life in Navarre, Spain [57], and in the USA from birth [30]). As the maximum concentration is reached in infants within a few days, it is possible that, in some infants, the protective concentration is not reached as quickly and that sub-neutralizing doses of antibodies circulate in the blood (or lungs) for some time, thus favoring ADE in epidemic periods in the event of an encounter with the virus. Similarly, neutralizing serum levels may not be maintained in all infants during the epidemic season due to the waning immunity conferred by nirsevimab. Pharmacokinetic pre-clinical studies should be performed in the presence and absence of viral infection to determine the effect of infection on antibody persistence; the half-life in uninfected and infected animals may differ [100]. With regard to the data on neonatal mortality in France during the immunization campaign presented above, it may be objected that mortality peaks are not sustained; if nirsevimab were causing excess deaths, there should be a sustained spike rather than a fluctuating pattern. This can be explained by the hypothesis of early ADE occurring only at sub-neutralizing concentrations in rare individuals due to inefficient biodistribution in the lungs in the first few days post-injection. Newborns affected by this ADE but who do not die immediately are less severely affected and are saved (to prove this, we would need access to data on ICU stays and the immunization status of patients).

### 6.2. Study of Nirsevimab Binding to FcγR In Vitro and Ex Vivo and of Possible ADE in Animals by Manufacturers

As ADE occurs for mAbs through binding of virus–antibody complexes to Fc receptors (FcγRs) on target cells, the EMA prescribes the methods to be used to assess this risk. In vitro and ex vivo cytotoxicity (ADCC), phagocytosis (antibody-dependent cellular phagocytosis (ADCP)), and complement activation must be explored. Pre-clinical in vitro and in vivo tests did not detect any effector functions of nirsevimab that might give rise to concern about ADE in humans. The analysis below will show why they were incomplete, as they were often carried out on mAbs similar to but different from nirsevimab; pre-clinical trials with nirsevimab were largely published after the product’s approval [120].

#### 6.2.1. In Vitro and Ex Vivo Studies

The conclusions of the EMA-EPAR [27] are contradictory when it comes to studying the effector functions of nirsevimab. According to the EMA, nirsevimab should exhibit normal Fc-mediated effector functions (complement activation, mediation of phagocytosis, antibody-mediated destruction of virus-infected cells, etc.). Nirsevimab does not prevent virions from attaching to cells. The EMA considers that effector functions are not part of nirsevimab’s mechanism of action [27] yet writes that the contribution of Fc-mediated effector functions to protection against RSV disease cannot be excluded—in the rapporteur’s opinion, pre-clinical data from the cotton rat model seem ambiguous in this respect [27] Mechanisms other than virus neutralization would be necessary for the therapeutic effect, such as viral clearance and killing of infected cells [4]. The EMA reiterates the importance of studying effector functions for their involvement in a possible ADE [27] (Fc effector functions are linked to the exacerbation of symptoms due to RSV [120,121]).

Before commercialization, the effector functions of nirsevimab were studied only in vitro or in cotton rats [20,122,123] and cynomolgus monkeys [70] and sometimes with a mAb different from nirsevimab. YTE mutations were shown to reduce FcγRIIIA binding by a factor of 2 (only with allotype F158), as well as antibody-dependent cell-mediated cytotoxicity (ADCC) activity by a factor of 100 [70]. This was confirmed in 2023 for ADCC in an in vitro study (on different human cell types). The EMA does not report any study of ADCC or the role of NK cells (which express only FcγRIIIA), which are nevertheless suspected of playing a primordial role in the balance between protection and pathology following the use of passive immunization [109,121]. Ex vivo (using sera from children participating in the nirsevimab clinical trial), the ADCC of NK cells mediated by FcγRIIIA (whose role is double-edged) is completely suppressed compared with that of palivizumab [120]. But it is known that the level of ADCC activity in vitro does not correlate with clinical symptoms in primary RSV infection, and the role of ADCC in the protection from or pathogenesis of RSV infection remains to be determined [109,120]. This reduction in ADCC by nirsevimab could influence the therapeutic effect. Indeed, the role of FcγRIIIa would be double-edged: it may be proinflammatory and contribute to RSV disease or it may be anti-inflammatory [102,103].

Nirsevimab binds to activating and inhibitory FcγRs and the result of these activating and inhibitory functions could vary according to the level of mAb present in the serum [120]. Ex vivo studies show that, at certain dilutions, nirsevimab may possess effector functions linked to the exacerbation of RSV symptoms [120], further underlining the importance of detailed pharmacokinetic studies. Dilution tests are performed on a pool of sera and do not sufficiently explore the influence of individual variations in nirsevimab concentration (the sera are collected 15 days after injection; analyses are performed on serum pools: 30 sera from treated participants are mixed, representing only 1.5% of the treated cohort). The results vary with serum dilutions and only stand out from controls for certain dilutions. According to the authors, effector functions cannot be predicted beyond 150 days post-injection. Nirsevimab promotes beneficial ADNP (neutrophil phagocytosis), but neutrophilic inflammation with infiltration is also associated with severe RSV disease. At certain dilutions, nirsevimab amplifies phagocytosis by macrophages, which can be double-edged and requires further research. Complement deposition compared to placebo is strongly increased at certain dilutions. However, the authors do not anticipate any deleterious effect on this effector function in vivo, without further clarification. Complement-dependent cytotoxicity (CDC) is not studied, which is unfortunate as complement can have a protective or pathogenic role in viral infections [120] and particularly in RSV infections [121]. Experimental methods proposed in the past (but overlooked) could be used to assess effector function of nirsevimab; as early as 1989, Gimenez describes an assay for evaluating ADE, which quantifies it by assessing the amount of virus released by a macrophagic line [71]. Gimenez [110] and Bournazos [103] also recall the gap between in vitro ADE assays and experimental in vivo systems.

Other specific studies should have been carried out in the presence of other antibodies, as neutralization and ADE activities are modified when two mAbs are mixed, and a synergistic ADE effect may occur in the presence of different antibodies [117]. The binding of mAbs to antigen can cause structural changes in the region where IgG binds to FcRn and also in its interaction with complement [71,119]; antigen binding could modify Fc effector functions [100]. No trials have been carried out in the presence of immune complexes of nirsevimab bound to F protein or virus; all trials are performed in the absence of antigen. Binding to FcγRs depends on the conformational state of IgG (open or closed) [5]; a possible conformational change in nirsevimab when bound to F antigen or virus could alter its binding to FcγRIII and consequently its effector functions and hence its therapeutic effect.

The role of the glycosylation of nirsevimab in Fc binding has not been explored. According to the EMA [27], nirsevimab is produced in Chinese hamster ovary (CHO) cells and has a glycosylation site in the Fc domain (Asn-306) like most mAbs; this glycosylation is of the complex type (EPAR specifies that it has been characterized without further precision). mAbs produced from Chinese hamster ovary cells can produce afucosylated mAbs [124]. Glycosylation may play a role in mAb binding to FcRγ and C1q [125,126]. In particular, non-fucosylated IgGs bind more strongly to human FcγRIIIA and ADCC assays may show enhanced cytotoxicity [72]. Glycosylation of the Fc region affects mAb stability and plays a role in CDC and ADCC functions by modulating binding to the Fcγ receptor [127]. Sialylation would not affect FcγRIIIa binding, but afucosylated IgG would have no ADCC activity [124,125]. Galactosylation of IgG1 positively influences C1q binding and CDC, and sialylation increases C1q binding of galactosylated IgG [125]. The glycosylation of nirsevimab should have been studied as well as its effect on the effector functions of the molecule [121].

mAbs with high antiviral activity could contribute to inflammation in the advanced stages of the disease, which is probably why they are not used therapeutically [109]. This possible immune disruption [77,113,114,115] was not assessed in the trials. Yet, there is a method for assessing the proinflammatory effect of immune complexes (IgG binding to FcγRI) on a macrophagic line [128].

**Figure 4:**
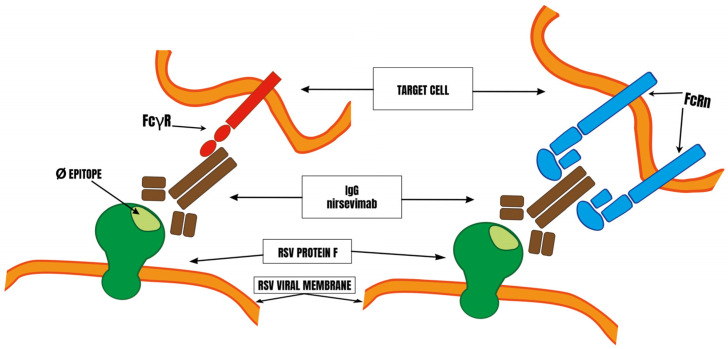
Relationship between nirsevimab function and ADE. Left: Strong binding to FcRn is able to modify other effector functions of nirsevimab that may be involved in its therapeutic effect (binding to FcγR of NK cells and complement components), particularly at low concentration. Right: FcRn is able to facilitate the entry of certain virus–antibody complexes into target cells (macrophages, pulmonary epithelial cells) at sub-neutralizing concentrations.

In vitro and ex vivo studies, albeit incomplete, show that nirsevimab has altered effector functions compared with other anti-RSV mAbs, particularly with regard to NK cytotoxic activity and phagocytosis by neutrophils and macrophages, as well as complement deposition (this is summarized in Figure 4). Increased binding to FcRn may disrupt the immune system; this risk has not been assessed. Many unknowns therefore remain, and it is a pity that some of the pre-clinical in vitro experiments were carried out only after the clinical trials and were incomplete.

#### 6.2.2. Animal Challenge Studies

During animal challenge studies, risk assessment should be carried out according to Munoz [129], as prescribed by the EMA: all cases of vaccine failure should be investigated for VAERD, viral infection should be confirmed by detection and quantification of the virus in specific sites (blood, upper and lower respiratory tracts, tissues), as well as characterization and sequencing of the virus; the immune response should be assessed and compared (in the present case mAb levels should be measured). Deposits and consumption of complement should be detected. Tissues obtained by autopsy should be evaluated for evidence of immunopathology. Although these post-challenge checks have not been carried out, the EMA concludes that ADE has not been observed in cotton rats, even at sub-effective doses [27].

In rat trials, it is necessary to use a mAb without YTE mutations, as these increase the affinity of FcRn in rats at neutral pH and suppress the protective effect of FcRn binding [4,120,122]. It is therefore difficult to extrapolate the results in these models to assess the absence of VAERD in humans. In the two studies with an antibody almost identical to nirsevimab (lacking the YTE mutations), pulmonary viral loads in cotton rats treated with the mAb and then challenged with RSV were greatly reduced compared with controls who had not received antibodies [4,120]. The authors conclude that these trials rule out the risk of ADE. However, we do not know what effect the increased affinity of YTE mutations would have on the viral loads. The reduction in viral loads does not depend on Fc effector functions [120], however, in humans, Fc effector functions are linked to exacerbation of RSV symptoms [5,101,121]. This calls into question the usefulness of ADE trials in rats. The effect of nirsevimab is being studied in cynomolgus monkeys, but they are not challenged with the virus, so the possible VAERD effect is not studied. No studies of ADE by immune system disruption have been carried out. No histopathological evaluation was carried out in rats in the trials (in particular to look for alveolitis and neutrophilic infiltration, which are the signs of ADE in this animal model). Moreover, the animals used were not suitable for characterizing ADE. The EMA stresses that as the rats used in the studies were mature, the translatability of the finding to highly immature infants is unknown [24]. The choice of animals to test for ADE is also problematic. In rats, effector functions are not necessary for protection against RSV infection, so the cotton rat model is not representative of human disease [120]. The animals usually used to study RSV (non-human primates, cotton rats, mice, and lambs) are semi-permissive to virus replication and experimental infection. Only chimpanzees are completely permissive to RSV. Cynomolgus macaques do not usually produce signs of disease unless infected with a high-titer inoculum. In the VAERD study in cynomolgus monkeys immunized with inactivated RSV, pulmonary pathology was not associated with increased viral replication, suggesting that the pathology was due to non-viral antigens. In cotton rats, too, the severity of alveolitis, used as a primary marker of VAERD with inactivated vaccine, depends on the response to non-viral antigens [130].

### 6.3. Clinical Trials

In clinical trials, tissues obtained by autopsy or biopsy should also be evaluated for immunopathology evidence; chest computed tomography (CT) has a high sensitivity for diagnosis of lower respiratory tract disease involvement [129] and should have been performed on participants with RSV LRTI. This has not been performed, however, the EMA concludes that ADE was not observed in clinical trials [27]. Moreover, respiratory infections not caused by RSV or not corresponding to the protocol have been neglected in clinical trials [20,23,26]. We found in the results of the immunization campaign that all-cause hospitalizations were not reduced compared with previous seasons.

## 7. Economic Benefits of Nirsevimab: Price and All-Cause Hospitalization Rates

The estimate of the comparative cost of nirsevimab immunization campaigns versus vaccination of mothers, based on product effectiveness, does not take into account all-cause hospitalizations. The price estimated to be profitable for society is lower than the price finally applied: Shoukat [131] proposes a price of CAD 290 to make nirsevimab cost-effective, and the Canadian NACI [132] a price of CAD 215 in a program aimed at all infants. However, the price set was higher in the USA (USD 495) [133] and was raised to USD 519.75 in April 2024 [134]. In France, the public price of nirsevimab was revealed in spring 2024 at EUR 401.80 [135]. It was not public when the 2023 campaign was launched [136]. In Germany, the price was EUR 1350.03 until May 15, 2024, when it was reduced to EUR 453.83 [137].

Neumann asks that the waning immunity and the possibility of emergence of resistant strains be taken into account in the real cost of nirsevimab (although Neumann, like other authors, relies solely on effectiveness against hospitalizations due to RSV). Immunized children are protected from RSV for only 6 months; nirsevimab-resistant strains are beginning to emerge [133]. All studies conclude that the immunization program is cost-effective only if applied to at-risk infants. A price reduction of 80–88% has been proposed to make the all-infant seasonal nirsevimab program with catch-up cost-effective [138]. Thus, taking into account only the burden of RSV infections, the price of nirsevimab would be too high to make immunization of all newborns cost-effective. The stated aim of nirsevimab immunization campaigns is to reduce the burden on hospitals. As no reduction in all-cause hospitalizations is observed in the age group undergoing nirsevimab immunization, the cost-effectiveness of mass campaigns will be even more questionable.

## 8. Conclusions

Nirsevimab was recommended in 2023 for all newborns and as a catch-up treatment for children during their first season of RSV circulation, with the aim of reducing the hospital burden due to infections. Based on clinical trials and published results from the first immunization campaign, it has been shown to be effective in reducing hospitalizations due to RSV (with the caveat that a large or unknown number of treated subjects were excluded in both clinical trials and post-marketing observational studies). But the overall burden on hospitals may not be reduced (all-cause hospitalizations are not reduced for infants and young children during the 2023–2024 season). This lack of net effect on the hospital’s overall burden may be attributed to the low share of RSV admissions in hospital admissions among under 5s. The results of the clinical studies show that the rare cases of hospitalization for LRTI due to RSV could have been more severe in treated participants and that the exclusion of a non-negligible proportion of treated participants could also have masked part of this effect. The responsibility of nirsevimab cannot be excluded for the deaths observed in fragile children. As regards the results of post-marketing immunization campaigns, the almost systematic exclusion of neonates hospitalized in the days following injection cannot rule out an early ADE effect. Some post-marketing observational studies exclude early cases (between 1 and 7 days) of RSV infection after treatment, and when data on these cases are available, they show a spike in early respiratory infections. Similarly, the small but significant increase in the number of newborn deaths between 2 and 6 days of age during the campaign in France is a warning signal of a possible ADE, even if no causal link can be established.

This ADE is well known as a consequence of inactivated vaccines and is due to antibodies against F protein, against which nirsevimab is directed. Nirsevimab has a very high affinity for RSV F protein and FcRn. Binding to FcRn protects nirsevimab from degradation but may also facilitate entry of the virus–antibody complex into cells expressing this receptor, particularly in the respiratory tract. This binding could be enhanced in certain individuals with acidic pH lung mucosa. The expansion of affinity for FcRn compared with the previously used mAb (palivizumab) could result in altered effector functions through binding to other FcγRs as well as to complement components. All these mechanisms have been proposed to explain ADE during RSV infection and this ADE has been shown in vitro on a monocyte line in the presence of anti-F antibodies. The early ADE effect in the days following injection may be due to sub-neutralizing concentrations of circulating anti-F antibody, which facilitates rather than blocks viral entry. Partial pharmacokinetic studies show this possibility. The lack of effect on all-cause hospitalizations could also be attributed to increased susceptibility to other infections, particularly respiratory. The immunodisruptive effect of IgG1 binding to FcRn could explain the increased susceptibility to other respiratory infections. Pre-clinical and clinical studies should therefore have paid particular attention to this risk. In pre-clinical studies, the FcγR-mediated effector functions were incompletely evaluated but showed that, at certain dilutions, nirsevimab may possess effector functions linked to the exacerbation of RSV symptoms. In clinical studies, RSV should have been systematically investigated in all participants, as should any immunopathological phenomena in hospitalized individuals (for RSV or not). In post-marketing studies, the immunization status of the subjects examined is not always known, and RSV testing is not systematically carried out in participants presenting a respiratory infection. The main bias of these studies lies in the exclusion of many subjects, particularly those who developed RSV infection within days of nirsevimab injection. Careful examination of the few data available suggests, however, that RSV infections are facilitated during this time interval (sub-neutralizing antibody concentrations may explain this phenomenon).

The strength of this study lies in the convergence of epidemiological and molecular arguments, suggesting the possibility of nirsevimab-induced ADE. If this ADE exists, it is of low frequency; given the shortcomings of published studies, it is therefore impossible to assert or evaluate its occurrence, and this is the main limitation of this work.

For future campaigns, immunization status should be systematically collected and all children presenting with ARI should be tested for RSV. Immunopathological phenomena should be explored in all those presenting with RSV infection after immunization with nirsevimab. mAb levels should be measured in serum and bronchoalveolar lavage (where available). In case of autopsy, diffuse inflammation of the alveoli and infiltration by neutrophils and eosinophils should be sought. In premature or unhealthy infants, it should be verified that sub-neutralizing levels do not persist longer than in healthy, full-term neonates. Long-term follow-up of treated children is needed to verify that sub-neutralizing concentrations circulating in the season following injection are not capable of causing ADE in the longer term, even if nirsevimab is protective in the short term. Nirsevimab should not be recommended for all neonates until these verifications have been carried out. These checks will enable us to confirm that it is scientifically and economically reasonable to continue recommending immunization of all infants and young children for the following seasons. This expensive product could be reserved only for at-risk children.

## Figures and Tables

**Table 1:** Summary results of clinical studies: efficacy of nirsevimab, argument in favor of ADE, biases.

	Absolute Reduction Risk RSV Hospitalization	Argument in Favor of ADE	Bias
Domachowske 2018 phase 1 healthy pre-terms [20]	Not evaluated	LRTI in treated group: 5 (5/69); LRTI in placebo group: 0 (0/16)	6.86% of treated participants are excluded
Griffin 2017 adults [21]	Not evaluated	URTI in 19% of treated participants; URTI in 9% of placebo group	No search for RSV carried out
Hammitt phase 2 [22]	1%	Treated participants hospitalized longer than placebo	8.1% of treated are excluded
Simöes [24]	2%	See [12,16]	2% of treated are excluded
Griffin 2020 pre-terms [26]	3.3%	Within 20–30 days after treatment LRTIs of all causes are equally frequent in both groups (early ADE not excluded)	5.8% of treated are excluded
Drysdale open label [22]	1.2%	More severe infections (any cause) in the treated group than in the placebo group	0.22% of treated are excluded. Investigator is sometimes the treating physician

In the phase 1b/2a infant and phase 1 adult trials, LRTIs due or not to RSV are mostly observed in the treated groups and not in the placebo group. Phase 3 and 2b trials are sometimes carried out outside periods of RSV circulation and therefore only imperfectly assess the possibility of ADE. The imbalance between placebo and treated groups may lead to a result biased in favor of efficacy [29]. Hospitalizations for LRTI due or not to RSV were rare and very slightly less frequent in the treated groups compared with placebo, but they could be more severe following treatment; in these rare cases, ADE could therefore explain these results. The high rate of children withdrawn from studies before analysis could distort efficacy results and the search for ADE. Nevertheless no ADE was observed by the EMA in clinical trials, despite greater signs of infection severity in treated groups and the frequent exclusion of infected subjects immediately after injection.

**Table 2:** Nirsevimab immunization coverage, effectiveness in post-marketing studies, argument in favor of ADE, and biases.

	Nationwide Immunization Coverage	Effectiveness RRR: Relative Risk Reduction Arr: Absolute Risk Reduction	Argument in Favor of ADE	Biases
USA-CDC Moline [39]	20%	RRR 90% against RSV hospitalization	77% of RSV hospitalizations observed within 7 days of injection	Symptoms < 7 days after injection are excluded
Luxembourg Ernst [41]	84%	Not evaluated	Impossible to assess	Immune status of participants not studied
France Paireau [45]	Unknown	RRR 74.4–80.6% against ICU admission due to RSV	Occurrence of PICU admission < 8 days after injection (or unknown): 45%	52% of cases excluded Case/control study 5/1
France Assad [42]	Unknown	RRR against RSV bronchiolitis 80%	No effectiveness during first 7 days	Case/control study 1/0.5 Non-representative population?
Spain Ares-Gomez [55]	>90%	RRR 82% against RSV hospitalization ARR of RSV LRTI admission: 1.6% RRR 66.2% against all-cause hospitalizations (comparison between seasons)	Nosocomial cases excluded (unknown number)	Non-comparable populations? No case-control possible Exclusion of seasons of low epidemic intensity and retention of the exceptional 2022–2023 season

From these results, we can conclude that nirsevimab is effective against RSV infections. However, in all studies, RSV infections occurring in the first few days following nirsevimab injection were not included. We cannot exclude that nirsevimab could in very rare cases cause facilitation/aggravation of RSV infection in the days following injection. On the other hand, nirsevimab does not reduce the rate of all-cause hospitalization in targeted populations, although hospitalization due to RSV is significantly reduced (however, efficacy and effectiveness may be less than advertised, given the large or unknown number of subjects excluded from clinical trials or observational studies).

## Data Availability

Not applicable.
